# A two-objective optimization of ship itineraries for a cruise company

**DOI:** 10.1007/s10288-023-00536-6

**Published:** 2023-02-24

**Authors:** Gianni Di Pillo, Marcello Fabiano, Stefano Lucidi, Massimo Roma

**Affiliations:** 1 ACTOR SRL, Start-Up of SAPIENZA University of Rome, Via Nizza 45, 00198 Rome, Italy; 2 Department of Computer, Control and Management Engineering “A. Ruberti”, SAPIENZA University of Rome, Via Ariosto 25, 00185 Rome, Italy

**Keywords:** Cruise shipping, Cruise itinerary optimization, Itinerary cost and attractiveness, Mixed Integer Linear Programming, Multiobjective optimization, 90B50, 90B06, 90C90

## Abstract

This paper deals with the problem of cruise itinerary planning which plays a central role in worldwide cruise ship tourism. In particular, the Day-by-day Cruise Itinerary Optimization (DCIO) problem is considered. Assuming that a cruise has been planned in terms of homeports and journey duration, the DCIO problem consists in determining the daily schedule of each itinerary so that some Key Performance Indicators are optimized. The schedule of an itinerary, i.e. the sequence of visited ports, the arrival and departure time at each port, greatly affect cruise operative costs and attractiveness. We propose a Mixed Integer Linear Programming (MILP) formulation of the problem with the objective of minimizing the itinerary cost due to fuel and port costs, while maximizing an itinerary attractiveness index. This latter is strongly related to the ports visited as well as to the overall schedule of the itinerary. Therefore the problem turns out to be a bi-objective optimization problem. We provide its solution in terms of Pareto optimal solution points. Each single objective MILP problem which arises is solved by using an exact algorithm, implemented in a commercial solver. We consider the day-by-day itineraries of a major luxury cruise company in many geographical areas all over the world. Here we report, as illustrative examples, the results obtained on some of these real instances.

## Introduction and literature review

In the last two decades, cruise shipping represented one of the most growing sectors of the shipping industry. According to Cruise Lines International Association (CLIA), the world’s largest cruise industry trade organization [see (CLIA 2021] for the latest report), since 1990 this sector has grown at an average annual passenger rate of 7.4% [see also (Cruise Market Watch 2020)]. Moreover, in the annual CLIA Global Market Report and State of Cruise Industry Outlook, the significant role played in international tourism by cruises is clearly evidenced: in 2019, the global cruise industry involved nearly 30 million passengers, creating jobs for 1.8 million people and contributing over $154 billion to the world economy. Of course the occurrence of the COVID-19 pandemic has dramatically hit the cruise industry, and all expectations for 2020 had to be drastically reconsidered [see e.g. (CLIA 2021] and (Notteboom et al. 2021, Chapter 1.5)). However the sector relies on a sound financial resilience, able to support the hope for better times in the next future. A revival of the sector is also expected in the medium term thanks to the adoption of new procedures with enhanced protocols related to passenger health and to the proposal of new attractive itineraries: according to *Seatrade Cruise News*,^1^ 27 new oceangoing cruise ships will be launched during 2021.

Cruise itinerary planning plays a fundamental role in the strategic decisions of a cruise company. Indeed, itineraries are announced in advance and they should attract booking as much as possible. Actually, itinerary planning is the last step in the decision making process of any cruise company which is usually characterized by the following three levels: (i) *the cruise fleet planning*, which is the highest level consisting in locating the ships in particular geographic areas in suited season windows so as to ensure the best weather conditions; (ii) *the ship deployment*, i.e. to decide which cruises must be planned in the chosen areas in terms of embarkation port and disembarkation port (named turnaround ports or homeports) and the cruise duration; (iii) *the day-by-day itinerary planning*, i.e., given the turnaround ports, to determine the sequence of intermediate ports (named ports of call or transit ports) to be visited by a ship and the arrival and departure time at each port. Note that there are turnaround ports which can also act as transit ports.

In a previous paper (Di Pillo et al. 2021), we have considered the second issue, namely the Cruise Itinerary Optimal Scheduling problem, aiming at determining a scheduling of cruises with the objective to maximize the revenue provided by a given ship placed in a specified maritime area, in a selected season window. Here we focus on the third issue, aiming at determining an optimal sequence of ports for the daily itinerary planning, minimizing the overall costs while maximizing customer satisfaction and taking into account several constraints on the itinerary design. In particular, we consider the cruise luxury market and this implies additional constraints usually not required by the cruise mass market. In fact, this latter usually offers to customers itineraries which are loops starting and ending at the same turnaround port and they are often repeated on week basis. Conversely, since customers of the cruise luxury market are usually returning customers, new itineraries, different from those already tried out, must be frequently proposed [see e.g. (Barron and Greenwood 2006)] and in many cases they are one-way itineraries (starting and ending turnaround ports do not coincide). Of course, in the ship deployment phase, the turnaround ports are usually chosen close to international airports and on the basis of infrastructures and services of the ports. Transit ports in the day-by-day itinerary planning phase are selected on the basis of a number of factors [see e.g. (Sigala 2017)] that go beyond port geographic location and availability of good facilities. Itinerary design is a critical issue for the success of a cruise since it strongly affects customers’ choice and hence it has a great impact on the occupancy rate of a cruise ship [see (Lee and Ramdeen 2013; Jeon et al. 2019)]. Different sequences of the visited ports usually result in different logistic organization, possibly improving customer satisfaction. Moreover it is very important to note that, as clearly pointed out in (Rodrigue and Notteboom 2013), customer choice of a cruise is based on the “overall appeal” of an itinerary. On one hand, this latter is certainly related to the attractiveness of the port cities visited, but on the other hand, it depends on the overall schedule of the itinerary and its operational conditions. Quoting from (Rodrigue and Notteboom 2013): “*the cruise industry sells itinerary, not destinations*”.

Therefore the Day-by-day Cruise Itinerary Optimization (DCIO) problem to be solved in the third level of the decision process of a cruise company involves many different significant aspects to be taken into account. This makes the problem really challenging both from the modellistic and computational viewpoint. Nevertheless, literature dealing with quantitative methods in the cruise company decision making processes and, in particular, in itinerary design is very limited. In the systematic review reported in (Papathanassis and Backmann 2011), the authors clearly evidence the scarcity of research on cruise shipping and this is due both to its domain’s niche status and to a wide fragmentation which arises from the interdisciplinary nature of cruise studies. In particular, by observing the conclusions reported in Table 2 of (Papathanassis and Backmann 2011), among the papers considered (published between 1983 and 2009) only 31% of them are quantitative research papers and only 6% are in the Engineering and Technology disciplinary domain. Even if this review refers to past years, anyhow in more recent years only few published papers actually use a mathematical approach. In particular optimization techniques are rarely used for efficiently solving problems related to cruise shipping management. This is true nevertheless the great growth of cruise market observed in the last years led to an increase of the dimension and the complexity of problems in hand. Most of the recent literature on cruise shipping is focused on Economics and Business Management, namely marketing strategies, revenue management, demand analysis [see (Cusano et al. 2017)]. To confirm this, see also the recent book (Dowling and Weeden 2017) which collects 35 papers providing a wide overview of the cruise industry covering a broad range of topics and issues. Even if the authors claim that the book has been written for a broad audience including planners and managers in the cruise industry, most of papers are focused on economic aspects (business models), environmental concerns (sustainable management), touristic issues (development of cruise tourism in particular regional areas), cruise safety and security (managing passenger health-related crisis). Until now, quantitative methods have been applied in maritime transport mainly dealing with freight transportation [see e.g. (Brouer et al. 2017; Gelareh and Pisinger 2011)] or passenger ferries [see e.g. (Wang and McOwan 2000)] rather than cruise sector.

In particular, few papers are devoted to mathematical modelling the day-by-day itinerary planning and to use optimization methods for solving DCIO problem. We mention the paper (Asta et al. 2018) where a Mixed Integer Linear Programming formulation of the DCIO problem is provided aiming at determining itineraries which maximize the revenues and customer satisfaction, and minimize the overall costs; (Cho 2019) where an Integer Programming model is proposed as a reduced variant of the traveling salesman problem, aiming at maximizing passenger satisfaction; the paper (Mancini and Stecca 2018) which proposes a model which is a variant of the vehicle routing problem along with a matheuristic which enables to efficiently solve large instances; (Wang et al. 2017) where the DCIO problem is solved by first enumerating all sequences of transit ports and then arrival and departure times are determined by using dynamic programming so that net profit is maximized; (Yang et al. 2016) where the authors developed a model for determining the maximum passenger volume with minimum operating costs by using a genetic algorithm.

Other papers on cruise itinerary design report results of empiric researches or are based on heuristic approaches. See, e.g. (Lekaku et al. 2009) where the authors focus on the selection of criteria to be used by a cruise company for deciding itinerary ports; (Leong and Ladany 2001) where an heuristic approach is proposed and applied to instances from South-East area; the paper (Li et al. 2020) which is limited to an analysis of the characteristic of the itineraries proposed by a world cruise company; (Santos et al. 2021) where the authors show how the distribution of nautical distances between ports in Atlantic coast of the Iberian Peninsula and Mediterranean ports can be used for itinerary planning.

In this paper, we consider the DCIO problem aiming at determining the day-by-day itinerary in terms of transit ports and arrival and departure times, with the objective of minimizing the itinerary cost due to fuel and port costs, and maximizing an attractiveness index of the itinerary. This latter is related to the ports visited and the number of days spent at sea, i.e. without docking in a port. Therefore the problem turns out to be a bi-objective optimization problem. As to the first objective, the fuel consumption depends nonlinearly on the ship speed; the speed depends on the distances between the ports and the need to meet times for entering and leaving the port; the port cost depends on the port location and on the services provided. As to the second objective, it is evaluated by giving a rating to each port, to the days spent at sea (when the travel time between two successive ports exceeds 24 h) and to overnights in port. Operational constraints are due to minimum and maximum number of transit ports to be visited, to the allowable time windows for arrival and departure in the port, to minimum and maximum time of stay in each port, to the fact that some ports may be obliged or prohibited, or may be visited only in given days, to minimum and maximum number of days spent at sea, to minimum and maximum number of ports where the ship moors at anchor and not at the dock. In particular, as we already mentioned, we refer to luxury cruises, implying several specific considerations to be taken into account, which lead to an increased difficulty of the problem.

We propose a bi-objective Mixed Integer Linear Programming (MILP) model for this problem. We provide its solution in terms of points of the Pareto frontier obtained by using two “a posteriori” methods (see, e.g., Miettinen (2012)): *the methods of weights* and *the method of constraints*. We solve all the single objective MILP which arise by using a commercial solver. Each Pareto optimal solution provides the optimal day-by-day itinerary, in terms of ports to be visited and times of arrivals and departures. This model has been experimented by a major luxury cruise company to design the day-by-day itineraries of their cruises in many geographical areas all over the world. Here we report, as illustrative examples, the results obtained on some of these real instances to show the computational viability of the proposed approach. In this regard, we highlight that we adopt an exact solution approach, rather than the use of some metaheuristic, even if for some large instances this may lead to long computing times. This is motivated by the fact that itinerary planning is performed years in advance, so that even a long computing time for some instances is admissible. Of course, if the computing time exceeds a CPU time threshold value, the computational run can be stopped early, providing an approximate solution of the problem with the corresponding optimality gap, so that its accuracy can be assessed.

This work has been developed within a project named *Magellano Project*, a joint project between ACTOR SRL, a Start-Up of SAPIENZA University of Rome and a major luxury cruise company (which we do not mention for the sake of privacy). The overall project involves the three levels of the decision making process of the cruise company.

The paper is organized as follows: in Sect. 2 the description of the DCIO problem is reported. In Sect. 3 we describe in detail the mathematical model developed. In Sects. 4 we describe how to use the model. In Sect. 5 we report some experimental results on real problem instances. Finally, some concluding remarks are drawn in Sect. 6.

## Problem description

In this section we report all the elements that characterize the DCIO problem, with a particular focus on a cruise company that operates in the luxury market class. The problem data are: a ship, a maritime area, the turnaround ports, a time period (defined by starting and ending date of the itinerary) and a set of transit ports of touristic interest in the area. The turnaround ports are assumed to be selected in the ship deployment second level of the company decision process.

Observe that there are two kinds of ports: those where the ship can moor at the dock, and those where the ship moors only at anchor. This partition is a specific feature of luxury cruise companies, which usually operate with small tonnage ships, embarking only hundreds of passengers and not thousands, as happens for the mass market class. Indeed a ship of small tonnage can enter small ports of great touristic interest, by mooring at anchor and debarking passengers by motor boats, which is not possible if the number of passengers is too large.

The design of a cruise itinerary first consists in selecting the transit ports and their sequence. Usually, in an itinerary, a transit port is visited just once: only a turnaround port can be visited twice in the case the cruise starts and ends at that port. Each day no more than one port is visited, due to the time required by the maneuvers for mooring, the time required for debarking and embarking passengers and the free time spent on shore excursions by passengers. Therefore, usually a cruise ship arrives in a port in the morning and departs in the evening. However it can be the case that an overnight is spent in a port, if there is an event that motivates a longer stay or if the port (or some neighborhood) is of great touristic interest and shore excursions may last more than one day. In this case, the ship will depart in the evening of the next day. Moreover, it is possible that, between one port and the next one in the sequence, the ship sails for more than one day, without intermediate mooring; we call these days “days at sea” and they could be included in an itinerary when the distance between two ports is very long as, for instance, in oceanic cruises. However, this may happen also if the company considers fruitful to keep passengers on board, so that they spend on board money that otherwise would spend on shore.

The overall aim of the cruise itinerary planning is twofold: to determine the sequence of ports along with arrival and departure time, aiming at minimizing the cruise itinerary cost, while maximizing its attractiveness. It is important to note that, typically, the more attractive is a cruise itinerary, the more its costs, therefore the problem has conflicting objectives. In the following, we detail how the overall cost is computed on the basis of a number of compound costs and how the attractiveness of an itinerary is determined.

The *itinerary cost* is obtained as the sum of fuel cost and port costs; in turns, fuel consumption depends nonlinearly on the distance between ports and on the speed at which the distances are covered. The cruising speed between two ports depends on the distance between the ports and on the time windows for leaving and entering the ports, being these latter usually prefixed by port operators. The port cost depends on the maneuvering cost in arrival and departure, and on the cost of the stay in the port which is given by a fixed and an hourly component.

The *itinerary attractiveness* is obtained as follows. At each port of the area a *Port Attractiveness Index (PAI)* is assigned on the basis of the score obtained by evaluating a list of port attributes. For the sake of brevity, we do not report here the complete list, but we only mention the most representative: *overall perception* (general reputation, is iconic, political stability, safety); *port features* (port infrastructures, distance to city center); *interests and activities* (cultural interest, natural interest, food and beverage interest, shopping possibilities, shore excursions options/variety); *exclusivity* (crowding level, exclusive cruise destination). Moreover, an index is assigned to possible one *Days At Sea (DASI)*. This is evaluated by giving a score to interests and activities that can be proposed to the passengers on board. Finally, we consider another index related to possible one *Overnight In Port (OIPI)*. All these indices have been evaluated by means of the scores assigned by people from the cruise company marketing office involved in the project. Based on their expertise, they provided us with accurate answers to a specific questionnaire we proposed. Hence, we define the itinerary attractiveness as weighted sum of these three indices PAI, DASI and OIPI.

We now summarize the basic requests which must be considered when dealing with the mathematical formulation of the DCIO problem:

1. a port cannot be visited twice in a cruise itinerary, except the embark port;
2. some ports of the considered maritime area could be banned in given days;
3. the visit at some ports is mandatory, i.e. they must be included in the cruise itinerary;
4. the visit of some ports is mandatory in prefixed days;
5. an overnight in a given port in a given day is planned;
6. a given day of the cruise must be a day at sea;
7. the days at sea can not be consecutive.

Moreover, the following data must be specified when designing a cruise itinerary:

1. the minimum and maximum number of transit ports visited in the cruise itinerary;
2. the maximum number of anchor ports included in the cruise itinerary;
3. for each port, the time windows for arrival and departure time in the port;
4. the minimum and maximum time of stay in each port;
5. the maximum number of days at sea included in the cruise itinerary.

Note that some constraints are relevant to luxury cruises: in particular, B2 bounds the number of possible anchor ports, due to the discomfort of disembarkation and embarkation by motor boats; B5 bounds the number of days at sea and, along with C7, aims at avoiding that the cruise could become boring.

## The mathematical model

In this section we describe the mathematical model we propose for solving the DCIO problem. In the following, we report all the elements of the model, i.e. the model input data, the decision variables, the objective functions and the constraints.

### The input data

The input data are divided into two groups: the *scenario data*, that are common to all problem instances for the same ship in the same maritime area, and the *instance data* that are peculiar to a particular instance of the problem.

#### The scenario data

The scenario of the model is defined by the following data:

- the set ${{\mathcal {P}}}$ of the ports of interest in the maritime area;
- the set ${{\mathcal {P}}}_A \subset {{\mathcal {P}}}$ of the anchor ports;
- the set ${{\mathcal {V}}}$ of the (discretized) operating cruising speeds of the ship;
- for $p,q \in {{\mathcal {P}}}, v \in {{\mathcal {V}}}$, the time required for sailing from port *p* to port *q* at speed *v* denoted by *t*(*p*, *q*, *v*);
- for $p,q \in {{\mathcal {P}}}, v \in {{\mathcal {V}}}$, the fuel cost for sailing from port *p* to port *q* at speed *v*, denoted by *c*(*p*, *q*, *v*);
- for $p \in {{\mathcal {P}}}$, the fixed and the hourly cost of the stay in port *p*, denoted by $c_f(p)$ and $c_h(p)$, respectively;
- for $p \in {{\mathcal {P}}}$, the cost of departure and arrival maneuvering in port *p*, denoted by $cm_d(p)$ and $cm_a(p)$, respectively;
- for $p \in {{\mathcal {P}}}$, the time duration of departure and arrival maneuvering in port *p*, denoted by $tm_d(p)$ and $tm_a(p)$, respectively;
- for $p \in {{\mathcal {P}}}$, the starting and ending time for the arrival time window at port *p*, denoted by $atw_s(p)$ and $atw_e(p)$, respectively; therefore, the arrival time window is $[atw_s(p) ~, ~ atw_e(p)]$;
- for $p \in {{\mathcal {P}}}$, the starting and ending time for the departure time window at port *p*, denoted by $dtw_s(p)$ and $dtw_e(p)$ respectively; therefore the departure time window is $[dtw_s(p) ~, ~ dtw_e(p)]$;
- for $p \in {{\mathcal {P}}}$, the minimum and maximum stay time in port *p*, denoted by *minstay*(*p*) and *maxstay*(*p*), respectively;
- for $p \in {{\mathcal {P}}}$, the attractiveness index PAI of port *p*, denoted by *a*(*p*);
- for $p \in {{\mathcal {P}}}$, the attractiveness index OIPI of one overnight at port *p*, denoted by *o*(*p*);
- the attractiveness index DASI of one day at sea, denoted by *ads*.

Note that, of course both PAI and OIPI indices depend on port *p*, while the last defined index (DASI), being related to the whole itinerary, does not depend on ports.

#### The problem instance data

The following data characterize a particular instance of the problem:

- the ordered set ${{\mathcal {D}}}=\{0, \ldots , N\}$ of the days of the cruise itinerary; $d\in {{\mathcal {D}}}$ denotes a day of the cruise; the cruise itinerary starts at day $d=0$ of the first embarkation and it ends at day $d=N$ of the last disembarkation;
- the embarkation and disembarkation turnaround ports of the cruise itinerary, denoted by $$p^e\in {{\mathcal {P}}}$$ and $$p^d \in {{\mathcal {P}}}$$, respectively; it may happen that $$p^e$$ and $$ p^d$$ coincide;
- the set ${{\mathcal {P}}}_T = {{\mathcal {P}}}\setminus \{p^e,p^d\}$ of the transit ports of interest;
- the set ${{\mathcal {P}}}_V \subset {{\mathcal {P}}}$ of the ports that must be visited by the cruise;
- the minimum and maximum number of transit ports to be visited by the itinerary cruise, denoted by *npmin* and *npmax*, respectively;
- the maximum number of anchor ports that can be visited by the itinerary cruise, denoted by $npmax_A$;
- the minimum and maximum number of days at sea allowed in the cruise itinerary, denoted by *mindas* and *maxdas*, respectively;
- the set ${{\mathcal {D}}}_S\subset {{\mathcal {D}}}$ of days $\{d^i ~: ~ d^i\in {{\mathcal {D}}}\}$, in which one day at sea must be planned, namely each day $d^i\in {{\mathcal {D}}}_S$ is such that more than 24 h must be spent at sea, starting from the embarking on the day $d^i$;
- the set ${{\mathcal {M}}}_V\subset {{\mathcal {P}}}\times {{\mathcal {D}}}$ of couples $\{(p^i,d^i), p^i\in {{\mathcal {P}}}, d^i\in {{\mathcal {D}}}\}$ of ports $p^i$ to be visited on day $d^i$;
- the set $\overline{{\mathcal {M}}}_V\subset {{\mathcal {P}}}\times {{\mathcal {D}}}$ of couples $\{(p^i,d^i), p^i\in {{\mathcal {P}}}, d^i\in {{\mathcal {D}}}\}$ of ports $p^i$ to be not visited on day $d^i$;

### The decision variables

Now we introduce the decision variables of the model, that result to be both continuous and integer (binary), so that we have a mixed integer problem.

- *x*(*p*, *q*, *v*) is a binary variable equal to 1 if in the itinerary cruise the ship covers the leg from port *p* to port *q* at speed *v*, equal to 0 otherwise;
- *y*(*p*) is a binary variable equal to 1 is the ship visits the port *p*, equal to 0 otherwise
- $y_d(p,q,d)$ is a binary variable equal to 1 if the ship departs from port *p* headed towards port *q* on the day *d*, equal to 0 otherwise;
- $y_a(p,d)$ is a binary variable equal to 1 if the ship arrives in port *p* on day *d*;
- *das*(*p*) is a binary variable equal to 1 if the ship arrives at port *p* having spent at least one day at sea, that is sailing for at least 24 h after the departure from the preceding port; equal to 0 otherwise;
- *ovn*(*p*) is a binary variable equal to 1 if the ship moors at port *p* at least 24 h, that is an overnight in port *p* is planned; equal to 0 otherwise;
- $t_d(p,q)$ is a continuous variable denoting the departure time of the ship departing from port *p* towards port *q*; $t_d(p,q)$ is expressed in terms of hours and hundredths of hour, in the interval $\{0,\dots , 24\times N\}$, thus increasing with the days;
- $t_a(p)$ is a continuous variable denoting the arrival time of the ship in port *p*; $t_a(p)$ is expressed in terms of hours and hundredths of hour, in the interval $\{0,\dots , 24\times N\}$, thus increasing with the days;
- $t_s(p)$ is a continuous variable denoting the stay time of the ship in port *p*; $t_s(p)$ is expressed in terms of hours and hundredths of hour, in the interval $\{0,\dots , 24\times (1+ovn(p))\}$.

### The objective functions

We now report the expressions of the objective functions used in our formulation of the DCIO problem. They depend on input data and variables previously introduced. As described in Sect. 2, we consider two objective functions: the itinerary total cost (denoted by *cost*) to be minimized and the itinerary attractiveness (denoted by *attr*) to be maximized. Both objectives result from the sum of different components, that we report in the following.

#### The itinerary cost

The objective function value *cost* of an itinerary is given by

$$\begin{aligned} cost = \hbox {fuelcost} + staycost + mancost, \end{aligned}$$

where

$$\begin{aligned} \hbox {fuelcost} = \sum _{p\in {{\mathcal {P}}}} \sum _{q\in {{\mathcal {P}}}} \sum _{v\in {{\mathcal {V}}}}c(p,q,v)x(p,q,v) \end{aligned}$$

is the total cost of fuel,

$$\begin{aligned} staycost = \sum _{p\in {{\mathcal {P}}}}\Big (c_f(p)y(p)+c_h(p)t_s(p)\Big ) \end{aligned}$$

is the total cost for staying in the visited transit ports and

$$\begin{aligned} mancost =\sum _{p\in {{\mathcal {P}}}_T}cm_a(p)y(p)+ cm_a(p^d) + {\sum _{p\in {{\mathcal {P}}}_T} cm_d(p)y(p)+cm_d(p^e)} \end{aligned}$$

is the total cost for maneuvering in arrival and departure at the visited ports.

#### The itinerary attractiveness

The objective function value *attr* is given by

$$\begin{aligned} attr=attrpts + attrdas + attrovn, \end{aligned}$$

where

3.1 $$\begin{aligned} attractpts=\sum _{p\in {{\mathcal {P}}}}a(p)y(p) \end{aligned}$$

is the attractiveness component due to the visited ports,

$$\begin{aligned} attractdas =ads\sum _{p\in {{\mathcal {P}}}}das(p) \end{aligned}$$

is the attractiveness component due to the days spent at sea and

$$\begin{aligned} attrovn = \sum _{p\in {{\mathcal {P}}}}o(p)ovn(p) \end{aligned}$$

is the attractiveness component due to the overnights spent in ports. Note that in (3.1) $p^e$ and $p^d$ have been included in the sum even if they contribute with a constant term.

### The constraints

In this section we describe the set of constraints which define the feasible set of the DCIO problem. They are subdivided into two groups: the *structural constraints* common to all problem instances, and the *operational constraints*, peculiar to a particular instance of the problem. In some constraints a parameter, denoted by *BigM*, is adopted to allow binary variables to turn constraints on or off.

#### Structural constraints

- Constraints ensuring that the cruise itinerary embarks and disembarks at the turnaround ports $p^e,\ p^d$:
  3.2 $$\begin{aligned} y(p^e)= 1, \ \ y(p^d)=1. \end{aligned}$$
- Constraint imposing that only one leg originates from the turnaround port $p^e$:
  3.3 $$\begin{aligned} \sum _{q\in {{\mathcal {P}}}} \sum _{v \in {{\mathcal {V}}}} x(p^e,q,v) = 1. \end{aligned}$$
- Constraint imposing that the ship departs from the turnaround port $p^e$ on the first day of the cruise:
  3.4 $$\begin{aligned} \sum _{q\in {{\mathcal {P}}}}y_d(p^e,q,0)=1. \end{aligned}$$
- Constraints imposing that on the first day of the cruise itinerary the ship cannot depart from ports different from the embarkation turnaround port $p^e$:
  3.5 $$\begin{aligned} \sum _{q\in {{\mathcal {P}}}}y_d(p,q,0)=0, \quad \hbox {for all} \quad p\in {{\mathcal {P}}}, \ p\ne p^e. \end{aligned}$$
- Constraints imposing that the ship cannot depart from the turnaround port $p^e$ on days different than the first day ($d=0$) of the cruise itinerary:
  3.6 $$\begin{aligned} \sum _{q\in {{\mathcal {P}}}}y_d(p^e,q,d)=0, \quad \hbox {for all} \quad d\in \{ 1,\dots ,N\}. \end{aligned}$$
- Constraints imposing that the ship cannot arrive to the turnaround port $p^e$ on days different from the last day ($d=N$) of the cruise itinerary:
  3.7 $$\begin{aligned} \sum _{p\in {{\mathcal {P}}}}y_d(p,p^e,d)=0, \quad \hbox {for all} \quad d\in \{0, 1,\dots ,N-1\}. \end{aligned}$$
- Costraints imposing that on the first day of the cruise itinerary ($$d=0)$$ the ship can not arrive to any port:
  3.8 $$\begin{aligned} y_a(p,0)=0, \quad \hbox {for all} \quad p\in {{\mathcal {P}}}. \end{aligned}$$
- Constraint imposing that the there is only one leg leading to the turnaround port $p^d$:
  3.9 $$\begin{aligned} \sum _{p\in {{\mathcal {P}}}}\sum _{v\in {{\mathcal {V}}}} x(p,p^d,v)=1. \end{aligned}$$
- Constraints imposing that on the last but one day of the cruise itinerary $(d=N-1$) the ship can not depart towards any port different from the turnaround port $p^d$:
  3.10 $$\begin{aligned} \sum _{p\in {{\mathcal {P}}}} y_d(p,q,N-1) = 0, \quad \hbox {for all} \quad q\in {{\mathcal {P}}}, q\ne p^d. \end{aligned}$$
- Constraints imposing that the ship cannot depart from the turnaround port $p^d$ unless $p^e = p^d$ and $d=0$, first day of the cruise itinerary:
  3.11 $$\begin{aligned} \sum _{q \in {{\mathcal {P}}}} y_d(p^d,q,d) = 0, \quad \hbox {for all} \quad d\in {{\mathcal {D}}}, d\ne 0. \end{aligned}$$
- Constraints imposing that the ship cannot depart on the last day $d = N$:
  3.12 $$\begin{aligned} \sum _{q\in {{\mathcal {P}}}} y_d(p,q,N)=0, \quad \hbox {for all} \quad p\in {{\mathcal {P}}}. \end{aligned}$$
- Constraints that set the variables *y*(*p*) to 1 if the port *p* with $p\ne p^e$ is visited, to 0 otherwise:
  3.13 $$\begin{aligned} y(p) = \sum _{q\in {{\mathcal {P}}}}\sum _{v\in {{\mathcal {V}}}} x(q,p,v), \quad \hbox {for all} \quad p\in {{\mathcal {P}}}, \ \ p\ne p^e. \end{aligned}$$
- Constraints relating the variables $y_a(p,d)$ and *x*(*q*, *p*, *v*):
  3.14 $$\begin{aligned} \begin{array}{rcl} \displaystyle \sum _{d\in {{\mathcal {D}}}}y_a(p,d) &{}=&{} \displaystyle \sum _{q\in {{\mathcal {P}}}}\sum _{v\in {{\mathcal {V}}}} x(q,p,v), \quad \hbox {for all} \quad p \in {{\mathcal {P}}}, \\ \displaystyle y_a(p^d,N) &{}=&{} \displaystyle \sum _{q \in {{\mathcal {P}}}} \sum _{v \in {{\mathcal {V}}}} x(q,p^d,v). \end{array} \end{aligned}$$
- Constraints on the variables *y*(*p*, *q*, *d*) imposing that in any given port, in any given day, at most one departure is possible:
  3.15 $$\begin{aligned} \sum _{q \in {{\mathcal {P}}}} y_d(p,q,d) \le 1, \quad \hbox {for all} \quad p\in {{\mathcal {P}}} \quad \hbox {and for all} \quad d\in {{\mathcal {D}}}. \end{aligned}$$
- Constraints imposing that any given port cannot be visited more than once during the cruise itinerary:
  3.16 $$\begin{aligned} \sum _{d\in {{\mathcal {D}}}} y_a(p,d) \le 1, \quad \hbox {for all} \quad p\in {{\mathcal {P}}}. \end{aligned}$$
- Constraints defining the arrival time of the ship in port *p*:
  3.17 $$\begin{aligned} t_a(p) = \displaystyle \sum _{q\in {{\mathcal {P}}}} t_d(q,p)+ & {} \displaystyle \sum _{q\in {{\mathcal {P}}}} \sum _{v\in {{\mathcal {V}}}} \Big ( t(q,p,v) + tm_d(q) + tm_a(p) \Big )x(q,p,v), \nonumber \\{} & {} \qquad \qquad \qquad \quad \hbox {for all} \quad p\in {{\mathcal {P}}}. \end{aligned}$$
  Note that, by this constraint, it results $t_a(p) = 0$ if the port *p* is not visited.
- Constraints defining the stay time of the ship in port *p*:
  3.18 $$\begin{aligned} \begin{array}{rcl} \displaystyle t_s(p^e)&{}=&{}0, \qquad t_s(p^d)=0, \\ t_s(p)&{}=&{} \displaystyle \sum _{q \in {{\mathcal {P}}}}t_d (p,q) - tm_a(p), \quad \hbox {for all} \quad p\in {{\mathcal {P}}}_T. \end{array} \end{aligned}$$
- Sequencing constraints on the departure times:
  3.19 $$\begin{aligned} \begin{array}{rcl} &{}&{}t_d(p,q) + \displaystyle \sum _{q \in {{\mathcal {P}}}}\sum _{v \in {{\mathcal {V}}}} (tm_{d}(p) + t(p,q,v) + tm_{a}(q))x(p,q,v) - \sum _{r\in {{\mathcal {P}}}}t_d(q,r) \\ &{}&{} \qquad \le BigM \left( 1-\displaystyle \sum _{q \in {{\mathcal {P}}}}\sum _{v \in {{\mathcal {V}}}} x(p,q,v)\right) , \quad \hbox {for all} \ p\in {{\mathcal {P}}}_T \ \hbox {and for all} \ q\in {{\mathcal {P}}}_T. \end{array}\nonumber \\ \end{aligned}$$
- Continuity constraints:
  3.20 $$\begin{aligned} \sum _{p\in {{\mathcal {P}}}}\sum _{v\in {\mathcal {V}}} x(p,q,v) = \sum _{r\in \mathcal{P}}\sum _{v\in {\mathcal {V}}} x(q,r,v), \quad \hbox {for all} \quad q\in {{\mathcal {P}}}_T. \end{aligned}$$
- Constraints required if the set ${{\mathcal {D}}}_S$ is not empty, i.e. if at least one day at sea is planned:
  3.21 $$\begin{aligned} \sum _{p\in {{\mathcal {P}}}}\sum _{q \in {{\mathcal {P}}}}y_d(p,q,d^i)=1, \quad \sum _{p\in {{\mathcal {P}}}}y_a(p,d^i+1)=0, \quad \hbox {for all} \quad d^i\in {{\mathcal {D}}}_S.\nonumber \\ \end{aligned}$$
  These constraints ensure that if the ship departs from some port on day $d^i\in {{\mathcal {D}}}_S$, it does not arrive in any port on day $(d^i+1 )$.
- Constraints imposing that the departure time of the ship occurs within the departure time window:
  3.22 $$\begin{aligned}{} & {} \sum _{d\in \{0, \dots , N-1\}}\!\!\!\!\!\!\!\!\!\!(dtw_s(p)+24d)y_d(p,q,d)\le t_d(p,q) \nonumber \\{} & {} \qquad \le \sum _{d\in \{0,\dots , N-1\}}\!\!\!\!\!\!\!\!\!\!(dtw_e(p)+24d)y_d(p,q,d), \end{aligned}$$
  for all $p, q\in {{\mathcal {P}}}.$ Note that by this constraint it results $t_d(p,q) = 0$ if the leg (*p*, *q*) does not belong to the itinerary.
- Constraints imposing that the arrival time of the ship occurs within the arrival time window:
  3.23 $$\begin{aligned} \sum _{d\in \{1, \dots , N\}}\!\!\!\!\!\!(atw_s(p)+24d)y_a(p,d)\le t_a(p)\le \!\!\!\!\!\! \sum _{d\in \{1, \dots , N\}}\!\!\!\!\!\!(atw_e(p)+24d)y_a(p,d),\nonumber \\ \end{aligned}$$
  for all $$p\in {{\mathcal {P}}}.$$
- Constraints on the minimum and maximum stay time in each port:
  3.24 $$\begin{aligned} minstay(p)\sum _{q\in {{\mathcal {P}}}}\sum _{v\in {{\mathcal {V}}}}x(p,q,v) \le t_s(p) \le maxstay(p)\sum _{q\in {{\mathcal {P}}}}\sum _{v\in \mathcal{V}}x(p,q,v),\nonumber \\ \end{aligned}$$
  for all $$p \in {{\mathcal {P}}}_T$$.
- Constraint that sets the variable $das(p) = 1$ if the ship arrives at port *p* after sailing for more than 24 h (day at sea), and sets $das(p) = 0$ otherwise:
  3.25 $$\begin{aligned}{} & {} -BigM \ (1-das(p)) \le \sum _{q \in {{\mathcal {P}}}} \sum _{v \in {{\mathcal {V}}}} t(p,q,v)x(p,q,v) \nonumber \\{} & {} \qquad - 24.01\le BigM \ das(p), \end{aligned}$$
  for all $$p \in {{\mathcal {P}}}$$.
- Constraint that sets the variable $ovn(p) = 1$ if the ship moors at port *p* for more than 24 h (overnight in port), and set $ovn(p) = 0$ otherwise:
  3.26 $$\begin{aligned} -BigM \ (1-ovn(p)) \le t_s(p) - 24.01 \le BigM \ ovn(p), \end{aligned}$$
  for all $$p\in {{\mathcal {P}}}$$.

#### Operational contraints

- Constraint on the minimum and maximum number of transit ports included in the cruise itinerary:
  3.27 $$\begin{aligned} npmin\le \sum _{p\in {{\mathcal {P}}}_T} y(p) \le npmax. \end{aligned}$$
- Constraint on the transit ports to be visited in the cruise itinerary:
  3.28 $$\begin{aligned} y(p) =1 \quad \hbox {for all} \quad p\in {{\mathcal {P}}}_V. \end{aligned}$$
- Constraint on the transit ports to be visited on given arrival dates in the cruise itinerary:
  3.29 $$\begin{aligned} y_a(p,d)=1 \quad \hbox {for all} \quad (p,d)\in {{\mathcal {M}}}_V. \end{aligned}$$
- Constraint on the ports to be not visited on given arrival dates in the cruise itinerary:
  3.30 $$\begin{aligned} y_a(p,d)=0 \quad \hbox {for all} \quad (p,d)\in \overline{{\mathcal {M}}}_V. \end{aligned}$$
- Constraint on the maximum number of anchor ports included in the cruise itinerary:
  3.31 $$\begin{aligned} \sum _{p \in {{\mathcal {P}}}_A} y(p) \le npmax_A. \end{aligned}$$
- Constraints on the minimum and maximum number of days at sea in the cruise itinerary:
  3.32 $$\begin{aligned} mindas \le \sum _{p\in {{\mathcal {P}}}}das(p) \le maxdas. \end{aligned}$$

By letting $npmin = npmax = N-1$ in (3.27) it is possible to impose that at each day one port is visited, thus avoiding days at sea and/or overnights in port. Instead, by setting $npmin< npmax < N-1$ more freedom is given for days at sea and/or overnights in port. The transit port to be visited is usually refereed to a port of great attractiveness: if the port is to be visited on this day, it means that some event occurs in this day which could be of great interest for the cruise passengers. The transit ports to be not visited in given days are typically referred to some shore activities that are not allowed in these ports in these days; as an example usually cruise ships do not moors in Civitavecchia (the port of Rome) on Monday, when in Rome all museums are closed. Of course, note that to impose that a port has not to be visited in the whole cruise itinerary it is enough to remove these ports from the set of ports ${{\mathcal {P}}}$. Note that since disembarking and embarking by motor boats would result somewhat uncomfortable for the cruise passengers, a maximum number of allowed anchor ports is prefixed by (3.31).

Finally, it is worth specifying that by constraints (3.25), one day $d^i$ at sea is forced if $\sum _{p\in \mathcal{P}}das(p)=1$ and

$$\begin{aligned} \sum _{p\in {{\mathcal {P}}}}\sum _{q \in {{\mathcal {P}}}}y_d(p,q,d^i)=1, \qquad \sum _{p\in {{\mathcal {P}}}}y_a(p,d^i+1)=0; \end{aligned}$$

of course, in this case, it is required that the set $\{t(p,q,v), p,q \in {{\mathcal {P}}}, v\in {{\mathcal {V}}} \}$ contains enough time legs $t(p,q,v) \ge 24$.

### The bi-objective mixed integer linear programming problem

We can now formulate the DCIO problem as the optimization problem aiming at minimizing the objective function *cost*, while maximizing the objective function *attr* defined in Sect. 3.3. Therefore, if we denote by ${{\mathcal {Z}}}$ the feasible set of the problem, i.e. the set of decision variables which satisfy all the constraints, then the DCIO problem can be stated as the following optimization problem

3.33 $$\begin{aligned} \begin{array}{l} \min \Bigl \{ cost(z), -attr(z)\Bigr \} \\ \hbox {subject to }z \in {{\mathcal {Z}}}. \end{array} \end{aligned}$$

Recalling that the decision variables are both continuous and integer (binary) and observing that both objective functions and all the constraints are linear, problem (3.33) is a *Mixed Integer Linear Programming (MILP)* problem. Moreover, based on the fact that, as we already observed, the more an itinerary is attractive, the higher its cost problem (3.33) has conflicting objectives, i.e. it is a nontrivial bi-objective optimization problem (for an extensive treatment on Multiobjective Optimization see, e.g.(Miettinen 2012)).

### Some remarks on the optimization model

We remark that, for the sake of simplicity, the model presented here is a simplified version of the one actually developed. In particular, as an example, some of the features that have been omitted are the following:

- to impose that the visited ports belong to different countries, thus avoiding to pay the “cabotage tax” (tax concerning rights of a company from one country to trade in a different country);
- to consider two (or more) consecutive days at sea, with decreasing attractiveness; this is of interest, in particular, for oceanic cruises;
- to consider more than one overnight in a port; this is of interest for events lasting more than one day;
- to prevent that anchor ports are visited in consecutive days;
- to consider more than one transit port in the same day, even if this is of interest only for cruises in the class of expeditions;
- for some given ports $p\in {{\mathcal {P}}}$, to consider the departure time window $[dtw_s(p), dtw_e(p)]$ and the arrival time window $[atw_s(p), atw_e(p)]$ split on two successive days.

It is clear that including in the description of the model all the additional features would require much more room, while they are not strictly necessary for understanding the approach we propose for tackling the cruise itinerary optimization problem.

## The optimization procedure

We now describe the optimization procedure leading to the design of the cruise itinerary which minimize the overall cost, while maximizing its attractiveness, namely to solve the MILP problem (3.33). To this aim, let us denote by ${{\mathcal {F}}}$ the two dimensional *objective functions space* (*cost*, *attr*). As well known, a solution $z^* \in {{\mathcal {Z}}}$ of problem (3.33) is a point that maps itself on the *Pareto frontier* or *efficient frontier* in ${{\mathcal {F}}}$. The latter is defined as the set of points for which any feasible movement from $z^*$ which improves the function *cost* on ${{\mathcal {F}}}$, worsen the function *attr* on ${{\mathcal {F}}}$ and conversely. Moreover, we consider the *ideal objective values* $cost^*$ and $attr^*$, i.e. the optimal values of the two single objective optimization problems resulting by considering separately the two objective functions, namely

4.1 $$\begin{aligned} \begin{array}{rl} \min &{}cost(z) \\ \hbox {s.t.} &{} z \in {{\mathcal {Z}}} \end{array} \end{aligned}$$

and

4.2 $$\begin{aligned} \begin{array}{rl} \max &{}attr(z) \\ \hbox {s.t.} &{}z \in {{\mathcal {Z}}}. \end{array} \end{aligned}$$

Furthermore, let us denote by $z^*_c$ and $z^*_a$ the optimal points of problems (4.1) and (4.2), respectively, namely points belonging to ${{\mathcal {Z}}}$ such that it results $cost^*=cost(z^*_c)$ and $attr^*=attr(z^*_a)$. Then we define $attr^\#=attr(z^*_c)$ and $cost^\#=cost(z^*_a)$.

As well known, the two methods elective for locating points of the Pareto frontier of a bi-objective optimization problem are the *method of weights* and the *method of constraints* [see, e.g., (Miettinen 2012)]. In the *method of weights* (also called *scalarization method*) the bi-objective problem is transformed into the single objective optimization problem whose objective function is the weighted sum of the two objective functions. Namely, it consists in solving the problem

4.3 $$\begin{aligned} \begin{array}{rl} \min &{}(1-w) \cdot cost(z) - w \cdot {\zeta } \cdot attr(z)\\ \hbox {s.t.} &{} z \in {{\mathcal {Z}}}, \end{array} \end{aligned}$$

where $w\in [0,1]$ is a suited weight. The scalar $\zeta >0$ is introduced in order to balancing the two objectives which can even differ of some order of magnitude, depending on the particular instance. Of course, setting $w = 0$ we get the objective function values $cost^*, attr^\#$, while setting $w = 1$ we get the values $cost^\#, attr^*$. By letting $w \in (0, 1 )$ we get other points of the Pareto frontier. More precisely, the solution of Problem (4.3) is *weakly Pareto optimal*; it is *Pareto optimal* if the weighting coefficients are positive, i.e., $w\in (0,1)$.

The *method of constraints* ($\epsilon$-constraint method) consists in the minimization of one objective function and considering the other objective as constraint, imposing that it is bounded by some threshold value. Namely, the following two problems can be considered

4.4 $$\begin{aligned} \begin{array}{rl} \min &{} cost(z) \\ \hbox {s.t.} &{} z \in {{\mathcal {Z}}} \\ &{} attr(z) \ge {\overline{attr}} \end{array} \end{aligned}$$

and

4.5 $$\begin{aligned} \begin{array}{rl} \max &{}attr(z) \\ \hbox {s.t.} &{} z \in {{\mathcal {Z}}} \\ &{} cost(z) \le {\overline{cost}}, \end{array} \end{aligned}$$

where the bounds ${\overline{attr}}$ and ${\overline{cost}}$ are such that

4.6 $$\begin{aligned} attr^\# \le {\overline{attr}} \le attr^* \qquad \hbox {and} \qquad cost^* \le {\overline{cost}} \le cost^\#. \end{aligned}$$

In both cases, by varying the values ${\overline{attr}}$ and ${\overline{cost}}$, we get points of the Pareto frontier.

It is worthy noting that, as well known, the drawback of using the method of weights is that not all the Pareto optimal solutions can be found (unless the problem is convex). On the contrary, by means of the method of constraints it is possible to determine every Pareto optimal solution, regardless of the convexity of the problem [see Miettinen 2012].

## Experimental results on some illustrative instances

The approach we propose in this paper has been experimented by a luxury cruise company for defining cruise itineraries for different ships located in various geographical areas all over the world. Of course, here we have no room for reporting results concerning the whole set of itineraries of the areas considered. We report the results obtained on some instances in order to show the reliability of our approach and also its computational viability. As regards the latter issue, we highlight that the DCIO problem results to be a large scale MILP problem, whose dimension increases with the cardinality of the sets ${{\mathcal {P}}}$ and ${{\mathcal {D}}}$, i.e. the number of ports considered and the duration of the itineraries. This could lead to long computing time needed to solve the problem. However, in the framework of the decision making process of a cruise company, computing time does not represent a crucial issue, since itinerary design is performed a long time in advance, usually a couple of years. This motivated us to use a commercial MILP solver (rather than adopt a metaheuristic solution approach), possibly stopping solver iterations when the relative optimality gap is below a prefixed threshold value.

We coded the MILP model for the DCIO problem by using AMPL language (Fourer et al. 2003) and we used the GUROBI 9.1 solver (GUROBI Optimizer reference manual 2020). All the runs have been performed on a PC with an Intel Core i7-2600 3.40 GHz Processor and 16 GB RAM.

All the reported results are obtained by using the following parameters: as regards the parameter *BigM* used in constraints (3.19), (3.25) and (3.26), by evaluating the order of magnitude of the quantities involved, we set $BigM=10^3$. Moreover, we set $\zeta =10^3$ in (4.3), on the basis of the observed values of *cost*(*z*) and *attr*(*z*) in the considered instances. Finally, in order to obtain optimal solutions, the runs were stopped when the relative optimality gap satisfies $rel\_opt\_gap \le 10^{-9}$; however, if this should lead to large computational times, a less tight threshold can be adopted, still ensuring a good accuracy of the results.

As illustrative example of a real instance of the DCIO problem we consider cruises in the West Mediterranean maritime area, embarking at Barcelona (Spain), disembarking at Civitavecchia, the port of Rome (Italy), and lasting 7 days. The set of transit ports includes 106 ports of Spain, France, Monaco, and Italy located in this maritime area. As regards the port names, in the following we adopt the standard abbreviation from the *United Nations Code for Trade and Transport Locations* (UN/LOCODE Code List 2020-2),^2^ consisting in a combination of a 2-character country code and a 3-character location code (e.g. ESBCN stands for Barcelona, Spain).

All the scenario data listed in Sect. 3.1.1 which refer to each port $p\in {{\mathcal {P}}}$ are given. The set of speeds ${{\mathcal {V}}}$ covers the operational interval [10, 18] nautical miles/hour, discretized by a 0.5 step. The arrival time window is set to [06:00,10:00] a.m. and the departure time window to [06:00,10:00] p.m. As regards the stay time in port we set $minstay=8$ hours and $maxstay=16$ hours. Finally the index DASI is given.

As regards the problem instance data reported in Sect. 3.1.2, we have the following ones common to all the instances reported in the following: $N=7$ in the definition of ${{\mathcal {D}}}$, the turnaround ports $p^e$=ESBCN and $p^d$=ITCVV, the set of transit port ${{\mathcal {P}}}_T$ (which is not reported extensively for the sake of brevity) whose cardinality is 106, $npmax_{{\mathcal {A}}}=1$. All the remaining problem data, namely *npmin*, *npmax*, *mindas*, *maxdas*, the sets ${{\mathcal {D}}}_S$, ${{\mathcal {M}}}_V$ and ${\overline{{\mathcal {M}}}}_V$, are specified in correspondence of each instance. By default, we assume: $\mathcal{P}_V=\emptyset$, ${{\mathcal {D}}}_S=\emptyset$ and $\mathcal{M}_V={\overline{{\mathcal {M}}}}_V=\emptyset$.

The results are reported in terms of values of the objectives *cost* and *attr* at the optimal solution, namely corresponding the feasible solution which satisfy the prefixed optimality gap; the computing time (in seconds) required to get such a solution. Tables reporting cruise itineraries include the list of the legs of the cruise itinerary^3^, the speed at which each leg is traveled (in nautical miles/hour), the voyage time required (in hours), the duration of the arrival maneuver (in hours), the arrival hour (within the 24 h), the stay time at port (in hours), the departure hour (within the 24 h), the duration of the departure maneuver (in hours), the index denoting if the port of arrival is a dock (D) or an anchor port (A), and the attractiveness of the leg (i.e. related to the arrival port or to a day at sea or to an overnight at port). Fractions of hour are expressed in hundredths. All cost and attractiveness values are scaled by a factor which is unspecified to protect strategic corporate data.

### Instance 1

In the first instance we consider $npmin=npmax=6$, $mindas=maxdas=0$, and we solve Problem (4.3) determining points of the Pareto frontiers by means of the method of weights and the methods of constraints. It is worthy nothing that, to provide the cruise itinerary planner with a set of (Pareto optimal) points, rather than a single optimal point, has a great practical importance. In fact, the decision maker can choose among the itineraries corresponding to these points according to company preferences, ensuring optimality of the adopted solution.

#### Pareto frontier by the method of weights

**Fig. 1:**
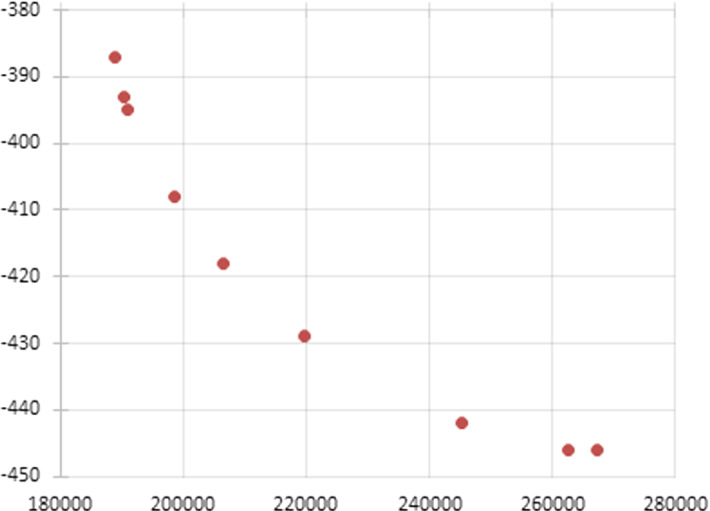
Points of the Pareto frontier obtained by the method of weights

We considered different values of the weight $w\in [0,1]$ obtained by discretizing the unit interval with step 0.1. The optimal function values of *cost*(*z*) and *attr*(*z*) obtained at each single objective minimizations are reported in Table 1, along with the elapsed CPU time. A plot of these points of the Pareto frontier is reported in Fig. 1.

**Table 1:** Method of weights: optimal point of each single objective minimization and CPU time (in seconds)

*w*	*Cost*	*attr*	CPU time
0.0	188,892	387	58.77
0.1	188,892	387	85.00
0.2	190,181	393	186.67
0.3	190,963	395	781.78
0.4	198,559	408	1284.39
0.5	206,488	418	1383.09
0.6	219,663	429	1321.41
0.7	245,228	442	2366.44
0.8	245,228	442	3708.39
0.9	262,593	446	3282.87
1.0	267,381	446	5995.00

Of course, the case $w=0$ corresponds to solve Problem (4.1) and it provides the ideal cost value $cost^*$ (obtained by minimizing the overall itinerary cost) and the value $attr^\#$ of the corresponding attractiveness; the case $w=1$ corresponds to solve Problem (4.2) and it provides the ideal value $attr^*$ of the attractiveness (obtained by maximizing the overall itinerary attractiveness) and the value $cost^\#$ of the corresponding cost. Note that the point obtained for $w=1$ is weakly Pareto optimal.

For the sake of brevity, we do not report here the cruise itinerary associated to each Pareto optimal point. However, in order to show some detailed itineraries, we report those obtained in the two extreme cases, namely $w=0$ and $w=1$. In particular, in Table 2 the optimal cruise itinerary obtained in the case $w=0$ is displayed.

**Table 2:** Optimal cruise itinerary for *Instance 1* corresponding to $w=0$ (minimization of the cost)

	*Port*	*Port*	*Speed*	*Voy.*	*Arr.*	*Arr.*	*Stay*	*Dep.*	*Dep.*	*D*	*Attr*
	*from*	*to*	*m/h*	*time*	*man.*	*hour*	*time*	*hour*	*man.*	*A*	
0		ESBCN						18.00	1.00	D	57
1	ESBCN	FRHYR	16.00	13.94	1.00	9.94	12.06	22.00	1.00	D	44
2	FRHYR	MCMCM	12.00	7.08	1.00	7.08	14.92	22.00	1.00	D	56
3	MCMCM	ITSMG	12.00	9.00	1.00	9.00	10.08	19.08	1.00	D	42
4	ITSMG	FRCLY	12.00	9.17	0.75	6.00	12.58	18.58	0.50	A	48
5	FRCLY	ITPTO	12.00	9.92	1.00	6.00	15.67	21.67	1.00	D	42
6	ITPTO	FRPVO	12.00	6.33	1.00	6.00	12.58	18.58	1.00	D	45
7	FRPVO	ITCVV	12.00	9.42	1.00	6.00				D	53

Since in this case the aim is to minimize the itinerary cost, from Table 2 it can be observed as the corresponding overall attractiveness relatively low is due to the inclusion in the selected itinerary of ports with a low PAI (see e.g. ITSMG and ITPTO). In Table 3 we report the optimal cruise itinerary obtained in the case $w=1$.

**Table 3:** Optimal cruise itinerary for *Instance 1* corresponding to $w=1$ (maximization of the attractiveness)

	*Port*	*Port*	*Speed*	*Voy.*	*Arr.*	*Arr.*	*Stay*	*Dep.*	*Dep.*	*D*	*Attr*
	*from*	*to*	*m/h*	*time*	*man.*	*hour*	*time*	*hour*	*man.*	*A*	
0		ESBCN						18.00	1.00	D	57
1	ESBCN	ESVLC	12.95	12.59	1.00	8.59	13.41	22.00	1.00	D	55
2	ESVLC	ESPMI	16.50	8.48	1.00	8.48	11.64	20.12	1.00	D	58
3	ESPMI	ESMAH	12.95	7.88	1.00	6.00	12.00	18.00	1.00	D	57
4	ESMAH	FRMRS	15.50	13.74	1.00	9.74	12.26	22.00	0.50	D	55
5	FRMRS	MCMCM	13.50	9.48	1.00	9.48	11.64	21.12	1.00	D	56
6	MCMCM	ITPTF	12.50	6.88	1.00	6.00	12.00	18.00	1.00	A	55
7	ITPTF	ITCVV	12.95	13.67	1.00	9.67				D	53

We can note that, as expected, all ports selected in this itinerary have a high attractiveness (greater than or equal to 53). Of course, the corresponding itinerary cost is significantly increased, too.

#### Pareto frontier by the method of constraints

Taking into account that the value of the objective *cost* is bounded from below by the values $cost^*=188{,}892$ and from above by the value $cost^\#=267{,}381$, we now solve Problem (4.5), for different values of ${\overline{cost}}$. More specifically, we consider values of ${\overline{cost}}\in [190{,}000, ~260{,}000]$ obtained by discretizing the interval with step 10,000. The optimal function values of *cost*(*z*) and *attr*(*z*) obtained at each single objective maximization are reported in Table 4, along with the elapsed CPU time.

**Table 4:** Method of constraints: optimal point of each single objective maximization and CPU time (in seconds)

${\overline{Cost}}$	*Cost*	*attr*	CPU time
190,000	188,925	387	350.45
200,000	199,563	408	1191.98
210,000	209,293	419	853.47
220,000	219,935	429	785.48
230,000	229,113	432	1213.28
240,000	238,113	436	1942.52
250,000	247,561	442	1658.09
260,000	258,686	444	2053.06

**Fig. 2:**
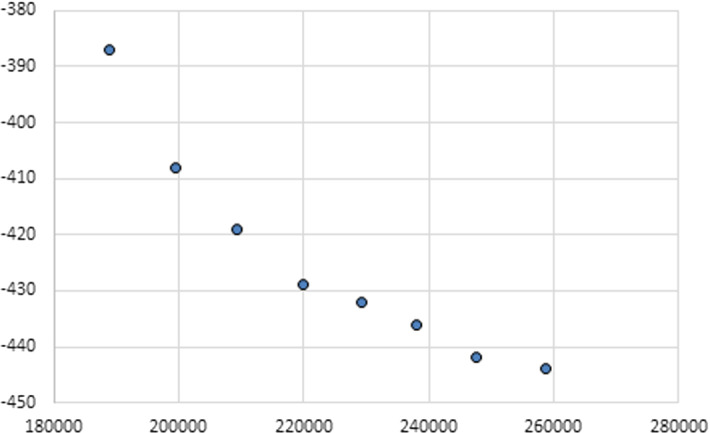
Points of the Pareto frontier obtained by the method of constraints

The fact that the higher port attractiveness, the higher port costs, is clearly observed. Indeed, a good value of the overall cruise attractiveness is obtained only if ${\overline{cost}}$ is not too low. A plot of the points of the Pareto frontier reported in Table 4 is displayed in Fig. 2.

Again, for the sake of brevity, we now detail in Table 5 the complete cruise itinerary for only one case, namely the one corresponding to ${\overline{cost}}=250{,}000$.

**Table 5:** Optimal cruise itinerary for *Instance 1* corresponding to ${\overline{cost}}=250{,}000$

	*Port*	*Port*	*Speed*	*Voy.*	*Arr.*	*Arr.*	*Stay*	*Dep.*	*Dep.*	*D*	*Attr*
	*from*	*to*	*m/h*	*time*	*man.*	*hour*	*time*	*hour*	*man.*	*A*	
0		ESBCN						18.00	1.00	D	57
1	ESBCN	ESPMI	12.95	10.19	1.00	6.19	12.54	18.73	1.00	D	58
2	ESPMI	ESMAH	11.00	9.27	1.00	6.00	12.00	18.00	1.00	D	57
3	ESMAH	FRMRS	16.50	12.91	1.00	8.91	9.09	18.00	1.00	D	55
4	FRMRS	FRAJA	13.50	13.70	1.00	9.70	8.30	18.00	1.00	D	51
5	FRAJA	MCMCM	12.95	10.35	1.00	6.35	14.48	20.83	1.00	D	56
6	MCMCM	ITPTF	12.00	7.17	1.00	6.00	12.00	18.00	1.00	A	55
7	ITPTF	ITCVV	13.00	13.62	1.00	9.62				D	53

It can be easily observed that the upper bound ${\overline{cost}}=250{,}000$ is high enough to allow including in the itinerary ports with high values of attractiveness.

We remark that the method of constraints can also be applied by considering Problem (4.5) varying the value of the bound ${\overline{attr}}$. We experimented this and since similar results are obtained, for brevity, we do not report them.

### Instance 2: visit of a particular port

In the preceding instance the port ITLIV is not included in any itinerary. However, since ITLIV (Livorno, Italy) is the basis for shore excursions to Pisa and Florence, both of great touristic interest, it could be important to impose that ITLIV is a port visited during the cruise itinerary. Therefore, we now use the same data of the previous *Instance 1*, but we set ${{\mathcal {P}}}_V=\,$ ITLIV. For the sake of brevity, we only report results obtained by the method of weights with an intermediate values $w=0.5$ in Problem (4.3). This should balance the two objectives *cost* and *attr*. Table 6 reports the obtained optimal point and the elapsed CPU time and Table 7 displays the corresponding complete cruise itinerary.

**Table 6:** *Instance 2* with $w=0.5$: optimal point and CPU time (in seconds)

*w*	*Cost*	*attr*	CPU time
0.5	212,515	417	1073.66

**Table 7:** Optimal cruise itinerary for *Instance 2* (${{\mathcal {P}}}_V=\{\textsf{ITLIV}\}$) corresponding to $w=0.5$

	*Port*	*Port*	*Speed*	*Voy.*	*Arr.*	*Arr.*	*Stay*	*Dep.*	*Dep.*	*D*	*Attr*
	*from*	*to*	*m/h*	*time*	*man.*	*hour*	*time*	*hour*	*man.*	*A*	
0		ESBCN						18.00	1.00	D	57
1	ESBCN	ESMAH	12.00	11.33	1.00	7.33	10.74	18.07	1.00	D	57
2	ESMAH	ITAHO	13.50	13.93	1.00	10.00	10.25	20.25	1.00	A	45
3	ITAHO	FRPVO	12.00	7.75	1.00	6.00	16.00	22.00	1.00	D	45
4	FRPVO	FRAJA	12.00	6.42	1.00	6.42	14.41	20.83	1.00	D	51
5	FRAJA	MCMCM	12.00	11.17	1.00	10.00	11.58	21.58	1.00	D	56
6	MCMCM	ITLIV	12.00	10.42	1.00	10.00	8.17	18.17	1.00	D	53
7	ITLIV	ITCVV	12.00	9.83	1.00	6.00				D	53

We can see that, as requested, the port ITLIV is visited during the cruise itinerary. In particular, this occurs on the 6th day of the itinerary, on the way to the final turnaround port ITCVV. However, to impose that ITLIV is among the visited ports leads to a decrease of the itinerary attractiveness since ports with a high PAI are not included to avoid high cost ports.

### Instance 3: one overnight in a particular port

Monte Carlo is well known for the motor race Grand Prix of Monaco, that was disputed on May 22–23, 2021. The next case considers the same data of *Instance 1* but now we add the request of an overnight in the port MCMCM on May 22, the 3rd day of the itinerary. This allows the cruise passengers to attend the race. Of course in this case the minimum stay time in MCMCM has been changed to 24 hours and the maximum to 48 hours. Note that in this case we have $npmin=npmax=5$ rather than 6, as in all previous cases. Once again, for the sake of brevity, we only report the results obtained by maximizing the itinerary attractiveness, namely by solving Problem (4.3) with $w=1$. Table 8 reports the obtained optimal point and the elapsed CPU time and Table 9 displays the corresponding complete cruise itinerary.

**Table 8:** *Instance 3* with $w=1$: optimal point and CPU time (in seconds)

*w*	*Cost*	*attr*	CPU time
1	232,267	440	474.86

**Table 9:** Optimal cruise itinerary for *Instance 3* (one overnight in MCMCM port) with $w=1$

	*Port*	*Port*	*Speed*	*Voy.*	*Arr.*	*Arr.*	*Stay*	*Dep.*	*Dep.*	*D*	*Attr*
	*from*	*to*	*m/h*	*time*	*man.*	*hour*	*time*	*hour*	*man.*	*A*	
0		ESBCN						18.00	1.00	D	57
1	ESBCN	ESMAH	11.00	12.36	1.00	8.36	9.64	18.00	1.00	D	57
2	ESMAH	FRMRS	16.50	12.91	1.00	8.91	10.26	9.17	1.00	D	55
3	FRMRS	MCMCM	14.50	8.83	1.00	6.00	37.88		1.00	D	56
4	Overnight at MCMCM										56
5	MCMCM	FRAJA	16.50	8.12	1.00	6.00	12.35	18.35	1.00	D	51
6	FRAJA	ITPTF	17.00	9.65	1.00	6.00	15.59	21.59	1.00	A	55
7	ITPTF	ITCVV	17.00	10.41	1.00	10.00				D	53

Observe that, as requested, one overnight at MCMCM port is scheduled in the 4th day of the itinerary and this affects the overall attractiveness of the itinerary cruise. In fact, a slight decrease of the attractiveness is obtained with respect to the optimal cruise itinerary corresponding to *Instance 1* (with $w=1$), from which this is derived.

### Instance 4: one day at sea on a particular day of the itinerary

As last instance we consider the same data of *Itinerary 1* with the following additional request: the itinerary must include a day at sea, starting with the departure in the evening on the 4th day and lasting during the whole 5th day. Of course, again we have $npmin=npmax=5$. Taking into account that, due to the reduced number of ports, we expect a reduced port cost and reduced attractiveness. Therefore, analogously to the previous instance, we consider Problem (4.3) with $w=1$, so that the itinerary attractiveness is maximized. Table 10 reports the obtained optimal point and the elapsed CPU time and Table 11 displays the corresponding complete cruise itinerary.

**Table 10:** *Instance 4* with $w=1$: optimal point and CPU time (in seconds)

*w*	*Cost*	*attr*	CPU time
1	283,672	411	393.25

**Table 11:** Optimal cruise itinerary for *Instance 6* (one day at sea at 5th day of the itinerary) with $w=1$

	*Port*	*Port*	*Speed*	*Voy.*	*Arr.*	*Arr.*	*Stay*	*Dep.*	*Dep.*	*D*	*Attr*
	*from*	*to*	*m/h*	*time*	*man.*	*hour*	*time*	*hour*	*man.*	*A*	
0		ESBCN						18.00	1.00	D	57
1	ESBCN	FRMRS	17.00	10.82	1.00	6.82	11.18	18.00	1.00	D	55
2	FRMRS	ESMAH	17.00	10.82	1.00	6.82	11.18	18.00	1.00	D	57
3	ESMAH	ESVLC	17.00	13.71	1.00	9.71	8.29	18.00	1.00	D	55
4	ESVLC	ESPMI	13.00	10.77	1.00	6.77	11.23	18.00	1.00	D	58
5	Day at sea										20
6	ESPMI	MCMCM	11.00	34.91	1.00	6.91	11.23	18.00	1.00	D	56
7	MCMCM	ITCVV	15.50	13.94	1.00	9.94				D	53

Of course, due to the low attractiveness which has been assigned in this case to the day at sea (20), the overall itinerary attractiveness is decreased with respect to that obtained in the previous *Instance 3*, even if its cost is significantly increased.

In order to give evidence of the large dimension of the problem in hand, we report the number of variables and the number of constraints which characterize the instances now considered: problems have typically around 62,600 variables of which around 2900 are continuous and the remaining are binary; the number of constraints varies between 18,850 and 22,040. Of course, the problem dimension increases as the number of ports considered $\vert \mathcal{P}\vert$ and the itinerary duration $\vert {{\mathcal {D}}}\vert$ increase. Therefore, solving the MILP problems corresponding to large instances could require a long computing time. However, as we already pointed out, usually this does not represent a serious drawback since the itinerary planning is designed a long time in advance.

## Conclusions

This paper deals with the day-by-day cruise itinerary planning for a ship operating in a given maritime area. It represents the lowest level of the decision making process of a cruise company in designing cruises to propose to their customers. In particular, we focused on luxury cruise market for which additional constraints must be taken into account, with respect to cruise mass market. Actually, the problem has a twofold objective: from one hand the aim is to minimize the overall itinerary cost and, from the other hand, the itinerary attractiveness should be maximized. These are two conflicting objectives, since the more attractive the itinerary, the higher the cost. Therefore, we formulated the DCIO problem as a bi-objective MILP problem, whose solution is provided in terms of Pareto optimal solution points. Actually, for the sake of clearness, in the paper a simplified version of the model experimented by a luxury cruise company is reported. However, the main features of the proposed approach can still be observed, avoiding discussions on technical details regarding additional specific requests.

As illustrative example, we report the optimal cruise itineraries for some instances in the West Mediterranean maritime areas. We highlight that the model has been used for defining cruise itineraries in many different geographical areas all over the world, considering many different parameter settings and particular additional requests. We showed that the model we propose allows the user to obtain the cruise optimal itinerary by using a commercial MILP solver. Of course, the optimization model we propose is to be intended as a decision support system for the company management, who is the only one deputed to refine, improve and finalize the cruise itineraries into the cruise catalog actually proposed to customers.

We believe that the revival of the cruise industry after the COVID-19 crisis should be also based on decision support systems like the one proposed in this paper, aiming at designing new and more attractive cruise itineraries, trying to contain costs.

## Footnotes

1 https://www.seatrade-cruise.com.

2 https://unece.org/trade/cefact/unlocode-code-list-country-and-territory.

3 For the sake of clearness, the explicit names of the ports listed in the tables which follow, are reported here. Ports of Spain: Barcelona (ESBCN), Porto Mahon (ESMAH), Palma de Mallorca (ESPMI), Valencia (ESVLC); ports of France: Ajaccio (FRAJA), Hyères (FRHYR), Marseille (FRMRS), Porto-Vecchio (FRPVO); ports of Italy: Civitavecchia (ITCVV), Livorno (ITLIV), Portofino (ITPTF), Porto Torres (ITPTO), San Michele di Ganzaria (ITSMG); port of Monaco: Monte Carlo (MCMCM).
